# IRF and STAT Transcription Factors - From Basic Biology to Roles in Infection, Protective Immunity, and Primary Immunodeficiencies

**DOI:** 10.3389/fimmu.2018.03047

**Published:** 2019-01-08

**Authors:** Trine H. Mogensen

**Affiliations:** ^1^Department of Infectious diseases, Aarhus University Hospital, Aarhus, Denmark; ^2^Department of Biomedicine, Aarhus University, Aarhus, Denmark; ^3^Department of Clinical Medicine, Aarhus University, Aarhus, Denmark

**Keywords:** IRF, STAT, interferon, antiviral, proinflammatory, primary immunodeficiency, genetics

## Abstract

The induction and action of type I interferon (IFN) is of fundamental importance in human immune defenses toward microbial pathogens, particularly viruses. Basic discoveries within the molecular and cellular signaling pathways regulating type I IFN induction and downstream actions have shown the essential role of the IFN regulatory factor (IRF) and the signal transducer and activator of transcription (STAT) families, respectively. However, the exact biological and immunological functions of these factors have been most clearly revealed through the study of inborn errors of immunity and the resultant infectious phenotypes in humans. The spectrum of human inborn errors of immunity caused by mutations in IRFs and STATs has proven very diverse. These diseases encompass herpes simplex encephalitis (HSE) and severe influenza in IRF3- and IRF7/IRF9 deficiency, respectively. They also include Mendelian susceptibility to mycobacterial infection (MSMD) in STAT1 deficiency, through disseminated measles infection associated with STAT2 deficiency, and finally staphylococcal abscesses and chronic mucocutaneous candidiasis (CMC) classically described with Hyper-IgE syndrome (HIES) in the case of STAT3 deficiency. More recently, increasing focus has been on aspects of autoimmunity and autoinflammation playing an important part in many primary immunodeficiency diseases (PID)s, as exemplified by STAT1 gain-of-function causing CMC and autoimmune thyroiditis, as well as a recently described autoinflammatory syndrome with hypogammaglobulinemia and lymphoproliferation as a result of STAT3 gain-of-function. Here I review the infectious, inflammatory, and autoimmune disorders arising from mutations in IRF and STAT transcription factors in humans, highlightning the underlying molecular mechanisms and immunopathogenesis as well as the clinical/therapeutic perspectives of these new insights.

## Introduction

Several decades of research uncovering the basic biology, regulation; and functions of the machinery for induction and responses to type I interferon (IFN) have paved the way for an understanding of a number of very diverse human diseases arising when one or more molecules in these pathways are defective. In this manner, the study of humans with primary immunodeficiencies (PID)s provides important understanding of specific protective immunity in humans, an insight that cannot always be gained by studying experimental animal models. Moreover, the study of individuals with defects in these pathways may teach us valuable lessons about principles of basic cell biology and infection immunology. Finally, detailed knowledge on the fundamental immunopathogenesis allows for rapid and specific diagnosis and not least for targeted treatments for individuals with these rare inborn errors of immunity.

Several PIDs have been associated to the defective expression or function of molecules belonging to innate immune signaling receptors or pathways (1, 2). These discoveries have been accelerated by the introduction of whole exome sequencing (WES) techniques. Moreover, with the advent of such sequencing methologies, an increasing number of monogenic diseases caused by gain-of-function (GOF) mutations have emerged (3). This expands the spectrum of PIDs to also include conditions characterized by autoimmunity and autoinflammation.

## Basic Structure, Signaling, and Biology of IRF and STAT Transcription Factors

### Recognition of Microbial Pathogens and Induction of IFNs by IRFs

**Figure 1:**
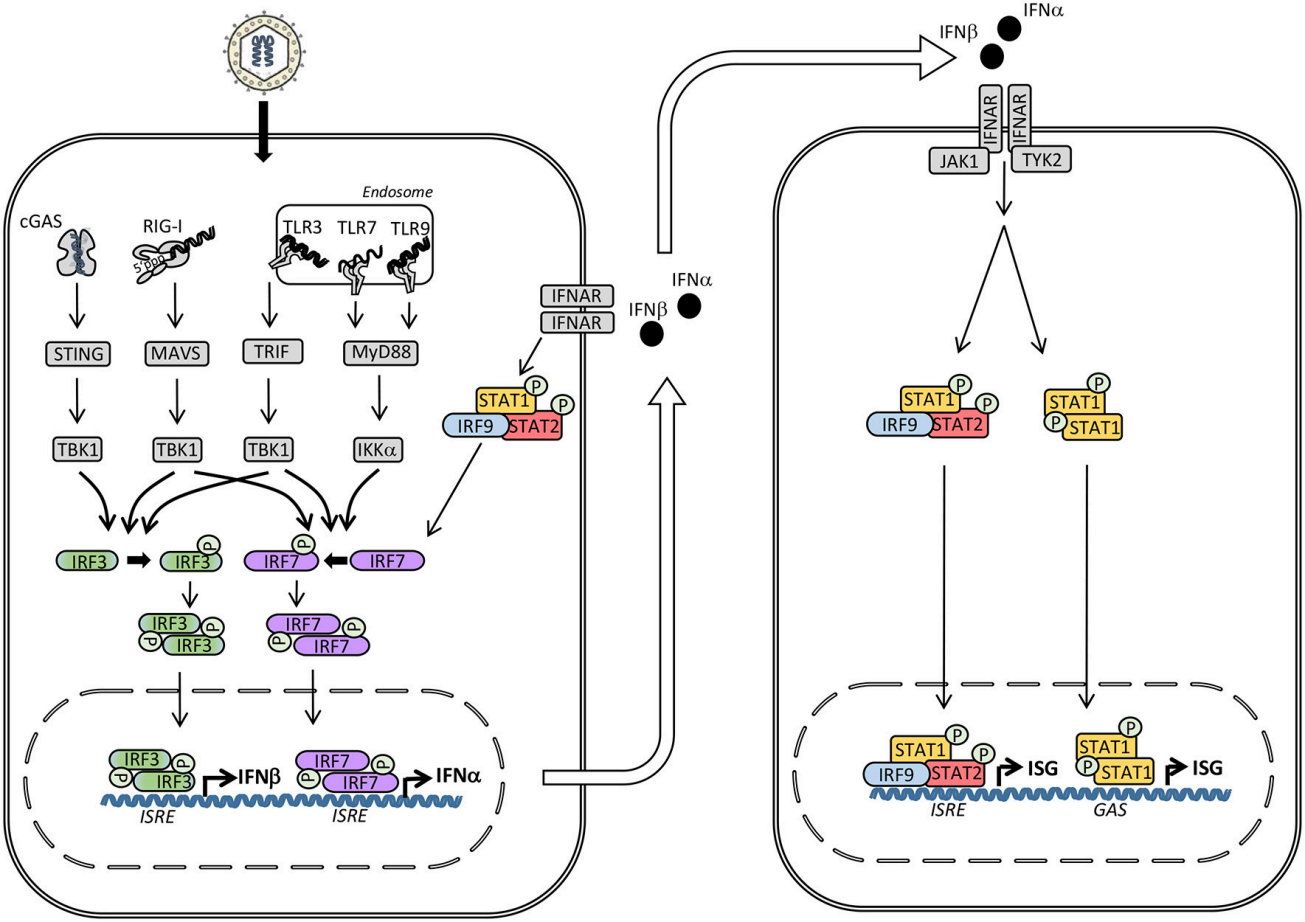
Induction of type I interferon (IFN) and signaling by the type I IFN receptor. The presence of microbial or self-nucleic acid in the cytosol or within the endosomal compartment activates pattern recognition receptors (PRR)s. RNA activates retinoic acid-inducible receptor (RIG)-I in the cytosol and Toll-like receptor (TLR)3 and TLR7 in the endosomal compartment, whereas DNA is sensed by cyclic GMP-AMP synthase (cGAS) in the cytosol and by TLR9 within endosomes. These events trigger signaling pathways through the adaptor molecules stimulator of IFN genes (STING), mitochondrial antiviral signaling protein (MAVS), TIR-domain-containing adapter-inducing interferon-β (TRIF), and Myeloid differentiation primary response (MyD)88 leading to phosphorylation and activation of the TANK binding kinase (TBK)1, which in turn phosphorylates the transcription factors IFN regulatory factor (IRF)3 and IRF7. Whereas IRF3 is constitutively present, IRF7 is expressed at low levels but may be secondarily induced by type I IFN. Phosphorylation of IRF3 and IRF7 leads to homodimerization, nuclear translocation, and expression of type I IFNs (IFNα and IFNβ) acting on neighboring cells with type I IFN receptors. Type I IFN binds to IFNα/β receptor (IFNAR)2 leading to recruitment of IFNAR1 and formation of a complex that activates the receptor-associated Janus-associated kinase (JAK)1 and tyrosine kinase (TYK)2 and subsequent tyrosine phosphorylation of STAT1 and STAT2. These activated transcription factors together with IRF9 form the heterotrimeric transcription factor IFN-stimulated gene factor (ISGF)3 complex which binds to IFN-stimulated regulatory elements (ISRE) in DNA. In addition, STAT1 homodimers form the IFN-γ-activated factor (GAF) complex which binds to IFNg-activated sequences (GAS). Altogether, these transcription factors induce a broad spectrum of IFN-stimulated genes (ISG)s that mediate the complex “antiviral state” of IFNs.

The innate immune system utilizes pattern recognition receptors (PRR)s to detect pathogen–associated molecular patterns (PAMP)s to mount protective immune responses, including production of cytokines and IFN (4). Production of type I IFN is induced following recognition of nucleic acids, mainly of foreign origin, but under certain pathological conditions deriving from the host. Different classes of PRRs are involved in induction of IFN, including membrane-associated Toll-like receptors (TLR)s, cytosolic RNA sensing retinoic acid inducible gene (RIG)-like receptors (RLR)s, and DNA sensors (4, 5). Each of these classes of PRRs activate IFN regulatory factor (IRF)s through unique adaptor molecules, known as TIR-domain-containing adapter inducing IFNβ (TRIF), mitochondrial antiviral signaling protein (MAVS) and stimulator of IFN genes (STING), respectively, to which IRF binds in order to become phosphorylated (6) (Figure 1).

### IRFs

In mammals nine different IRF family members have been described. All IRF proteins have a conserved amino-terminal DNA binding domain (DBD) with a helix-loop-helix structure and a motif containing five tryptophan residues. IRF-association domains (IAD)1 and 2 at the carboxyterminal region of IRFs mediate homodimeric and heterodimeric interactions with other IRF family members, transcription factors, and co-factors. Whereas IRF3 has restricted DNA binding properties, IRF7 exhibits broader DNA binding specificity, accounting for its capacity to induce several IFNα subtypes. The C-terminal signal response domain of IRF proteins contains several possible phosphorylation sites, of which phosphorylation at serine 386 for IRF3 or serine 477 and 479 at IRF7 are believed to represent the main activation sites (7). In addition, poly-ubiquitination of IRF7 at K63 by TRAF6 and the E2 enzyme complex Ubc13/Uev1A is required for IRF7 activation (8).

Transcriptional activation of the IFNβ promoter by IRF3/7 has been extensively studied (9, 10), and proceeds with phosphorylation of IRF3/7 by the kinase TKB1, leading to dimerization and translocation of IRF3/7 homo-or hetero-dimers to the nucleus together with other transcription factors, such as nuclear factor (NF)-κB and activator protein (AP)-1 (11). Assembly of the enhanceosome on the IFNβ promoter leads to histone acetylation and displacement of the nucleosome, hereby allowing initiation of IFNβ gene transcription. Importantly, NF-κB can cooperate in the induction of IFNs, at least at the IFNβ promoter, whereas IRFs, on the other hand, do not take part in induction of proinflammatory cytokines, although they may indirectly stimulate their synthesis (12, 13). IRF3 appears to be constitutively expressed in many cell types, residing in the cytoplasm in an inactive form, which upon upstream activating signals induces transcription of a set of early transcribed type I and type III IFN genes (mostly IFNβ, IFNα4, and IFNα1) (13, 14). IRF7 on the other hand, is a lymphoid transcription factor constitutively expressed only in B cells, monocytes and plasmacytoid dendritic cells (pDC)s (15). pDCs in particular express high levels of IRF7, while in most other cell types IRF7 is inducible from low levels of expression (16). Thus, early during infection IRF7 only has a minor contribution to the production of type I and type III IFNs. However, at later stages of infection, as more IRF7 is induced by type I IFNs, IRF7 induces the production of a delayed set of IFNα genes, including IFNα2, α5, α6, and α8, as well as IFNβ2 and IFNβ3 (14, 17, 18) (Figure 1). This generates a positive feedback loop, as type I and III IFNs induce more IRF7, thus leading to production of even more type I and type III IFNs, and this amplification loop is believed to play an important role in the generation of an immediate and potent response to virus infection (16).

### The Interferons

Type I IFNs were initially discovered as soluble factors mediating viral interference (19), and subsequent work allowed the cloning, sequencing, and functional characterization of this group of cytokines (11, 20–24). Whereas type I IFNs (IFNα and IFNβ) are predominantly expressed by innate immune cells, the functionally similar type III IFNs, (IFNλ1-4), discovered in 2003, are more restricted and primarily act on epithelial surfaces (17, 25). Finally, type II IFN (IFNγ), discovered in 1965, is being synthesized by Natural killer (NK) cells and T cells and exerts antiviral functions mainly by activating macrophages (25). Type I IFNs exert a broad range of antiviral activities, including induction of the classically described molecules dsRNA-activated protein kinase R (PKR) which inhibits the cellular translational machinery, 2′-5′-oligoadenylate synthetase (OAS)/RNAseL with the capacity to degrade foreign RNA, and Mx proteins that mainly restrict influenza virus through an itranuclear GTPase activity (11). Moreover, a wide range of IFN-inducible genes (ISG)s with diverse effects on antiviral defenses and cell proliferation and differentiation are induced and mediate the pleiotropic effects of type I (and III) IFNs (11).

### JAK-STAT Molecules

**Figure 2:**
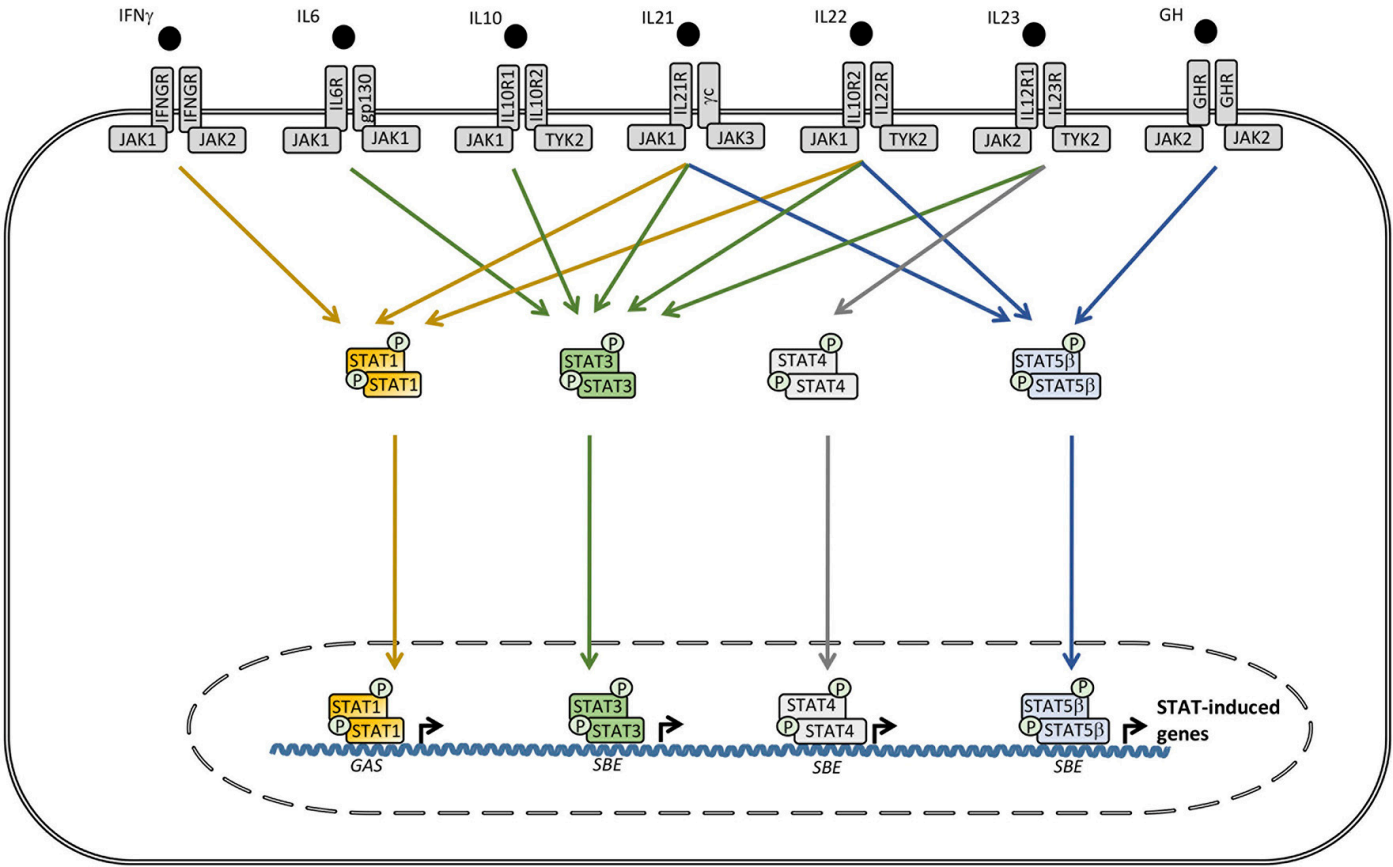
A wide range of cytokines, including interleukin (IL)6, IL10, IL21, IL22, IL23, IFNγ, and growth factors activate receptors utilizing different combinations of the tyrosine kinases janus activated kinase (JAK)1, JAK2, and tyrosine kinase (TYK)2 which trigger signaling pathways involving signal transducer and activator of transcription (STAT)1, STAT3, STAT4, and STAT5A/B. Phosphorylated STAT3 can homo- or heterodimerize with other STAT3 molecules or STAT1 or STAT5, respectively. STAT complexes modulate transcription of various genes, including increased IL6, IL10, IL17A/17F, IL22, transforming growth factor (TGF)β, and monocytic chemotactic protein (MCP)1 production, as well as decreased tumor necrosis factor (TNF)α, IL12, and IFNγ synthesis. SBE, STAT binding element. GAS, γ-IFN-activated sequence.

The evolutionarily conserved Janus kinase-signal transducers and activators of transcription (JAK-STAT) signaling pathway mediates responses to a number of important cytokines and growth factors (26) (Figures 1, 2). The specific responses to JAK-STAT signaling are therefore highly dependent on the cellular context and include proliferation, differentiation, migration, apoptosis, and cell survival (27). As a consequence, JAK-STAT pathways are involved in various physiological processes, including innate and adaptive immune responses, hematopoiesis, growth, and development (28). The identification of the JAK-STAT pathway was the result of seminal work performed by several scientists, including Darnell, Stark, and Kerr in the search for molecules mediating IFN-induced signaling (29). Four members of the JAK family (JAK1, 2, 3, and tyrosine kinase (TYK)2 and seven members of the STAT family (STAT1, 2, 3, 4, 5A, 5B, and 6) have been identified (28). JAKs belong to a class of tyrosine kinases characterized by containing both a catalytic domain and a kinase-like domain with autoregulatory function (29). By functional screening, these kinases were subsequently functionally linked to the transcription factors STATs (26). The overall structure of STAT proteins consists of coiled coil (CC) domain, a DNA binding domain (DBD), and an SH2 domain (29).

### JAK-STAT Signaling Downstream of the IFN Receptor

Type I IFNs act via the IFNα/β receptor (IFNAR)1 and IFNAR2, type III IFN signals through IFNLR1 and IL10R2, whereas IFNγ acts via IFNGR1 and IFNGR2 (11) (Figure 1). The binding of type I IFN induces formation of a receptor complex between IFNAR1 and IFNAR2, leading to activation of the receptor-associated JAK1 and TYK2 kinases (11, 22, 25). This is followed by recruitment and tyrosine phosphorylation of STAT1 and STAT2, which together with the transcription factor IRF9 form the heterotrimeric IFN-stimulated gene factor (ISGF)3 complex that binds to IFN-stimulated regulatory elements (ISRE) (26, 28, 29). In addition, phosphorylated STAT1 homodimers, termed IFN-γ-activated factor (GAF), are activated and in a similar manner induce transcription from γ-IFN activation sequence (GAS) (30) (Figure 1). Signaling from the type III IFN receptor is similar. In the case of type II IFN, binding of IFNγ induces homodimerization of IFNGR1 subunits and recruitment of IFNGR2 subunits, and this association induces phosphorylation and activation of JAK1 and JAK2 kinases, ultimately leading to phosphorylation of STAT1 to form the GAF complex as well as weak activation of the ISGF3 complex (11).

### STAT3 Signaling Downstream of Multiple Cytokine and Growth Factor Receptors

The STAT3 molecule was discovered by Akira et al. by purification and cloning and was found to bind to the interleukin (IL)6 responsive element of the acute phase response promoter (31). Simultaneously STAT3 was described by another group as a DNA binding protein downstream of the epidermal growth factor receptor (32). Pathways involving STAT3 activation are triggered by a number of cytokines and growth factors (27) (Figure 2). Receptor binding of the ligand leads to recruitment of intracellular JAK2 and TYK2, resulting in specific phosphorylation on STAT3 at tyrosine 705, allowing dimerization, nuclear translocation, and transcriptional activation of target genes (27). Phosphorylated STAT3 primarily homodimerize but also has the capacity to heterodimerize with STAT1 and STAT5, thereby inducing differentiated transcriptional programs (Figure 2). The 770 amino acid STAT3 molecule may be further post-translationally modified by phosphorylation, methylation, and acetylation, contributing to functional regulation (27, 28). The set of genes induced by actvated STAT3 is extraordinarily high and diverse, including IL10, IL17A/F, IL22, IL26, transforming growth factor (TGF)β, IL6, and monocytic chemotactic protein (MCP) 1. In contrast, pro-inflammatory mediators, such as tumor necrosis factor (TNF)α, IFNγ, and IL12, are downregulated through STAT3-mediated signaling pathways (27) (Figure 2).

## Diseases Associated with Defective IRF Signaling

**Figure 3:**
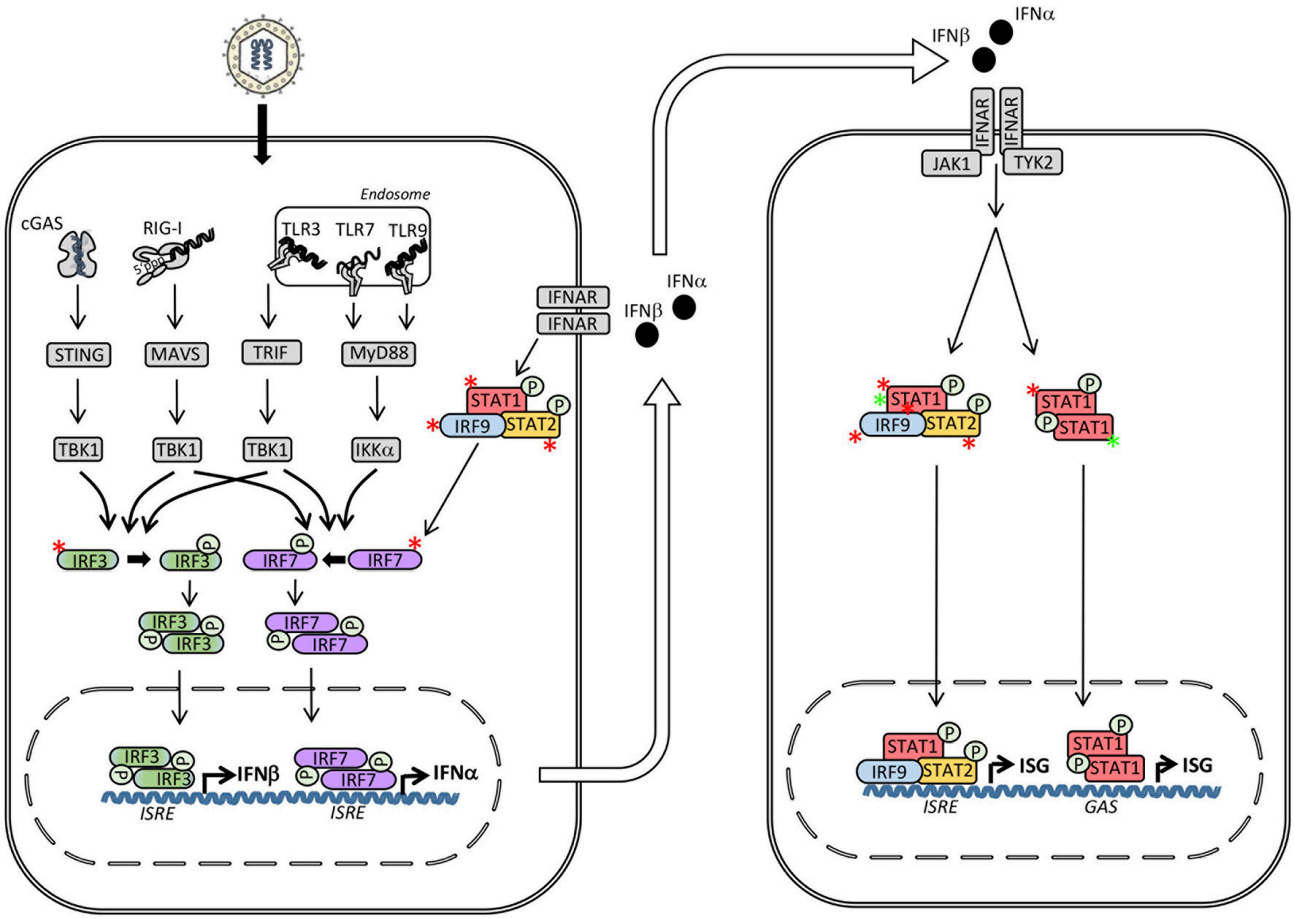
Inborn errors of immunity in interferon (IFN) regulatory factors (IRF)s and signal transducers and activators of transcription (STAT)s may lead to either loss-of-function (LOF) (red asterisk) or gain-of-function (GOF) (green asterisk) of the molecule and result in different primary immunodeficiencies (PID)s and infectious phenotypes. Within the IFN inducing signaling pathways defects in IRF3, IRF7, IRF9, and IRF8 result in herpes simplex encephalitis (HSE), severe influenza, and Mendelian susceptibility to mycobacterial disease (MSMD), respectively. Defects downstream of the type I IFN receptor in STAT1, STAT2 or IRF9 cause MSMD, susceptibility to measles, and severe influenza, respectively. In contrast, STAT1 GOF causes chronic mucocutaneous candidiasis (CMC). ISRE, IFN stimulated response element; GAS, γ-IFN-activated sequence.

**Figure 4:**
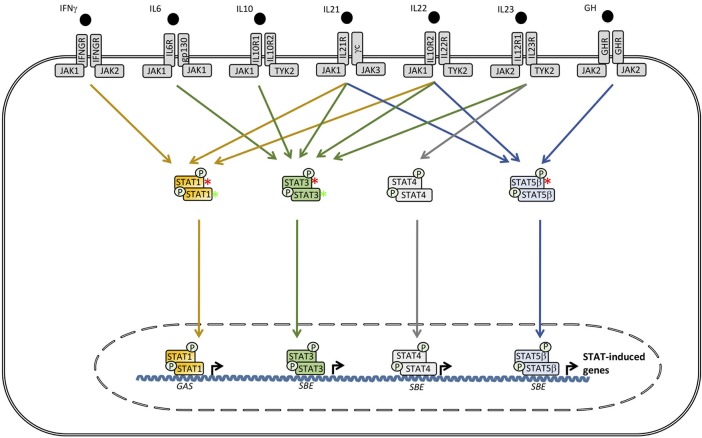
Defects downstream of various receptors utilizing signal transcducer and activator of transcription (STAT)1, STAT3, or STAT5B cause Mendelian susceptibility to mycobacterial disease (MSMD), hyper-IgE syndrome (HIES) and growth hormone insensitivity syndrome (GHIS), respectively. In contrast, STAT1 GOF causes chronic mucocutaneous candidiasis (CMC), whereas, STAT3 GOF leads to an autoinflammatory condition with hypogammaglobulinemia, lymphoproliferation, and a broad infectious phenotype. SBE, STAT bindinf element; GAS, γ-IFN-activated sequence.

In the second part of this review, presently known PIDs associated with defects in IRF and STAT transcription factors are described, including the clinical presentation, infectious phenotype and the genetic and immunological basis of disease. An overview of individual genetic defects and associated PIDs is given in Table 1, and the signaling pathways and transcription factors affected by either defective or excessive function of IRFs and STATs are illustrated in Figures 3, 4.

**Table 1:** Mutations in IRFs and STATs, functional impact, and associated PID/infectious phenotype.

**Gene**	**Inheritance**	**Allele**	**PID and infectious phenotype**
IRF3	AD/IP	LOF	Herpes simplex encephalitis (HSE)
IRF7	AR	LOF	Severe influenza
IRF8	AR	LOF	Mendelian susceptibility to mycobacterial infection (MSMD)
IRF9	AR	LOF	Severe influenza
STAT1	AD/AR	LOF	MSMD, HSE, fungi
STAT1	AD	GOF	Chronic mucocutaneous candidiasis (CMC), progressive multifocal leukoencephalopathy (PML)/JC virus
STAT3	AD	LOF	Hyper-IgE syndrome (HIES), *Staphylococcus aureus, Candida albicans*, aspergillus, EBV
STAT3	AD	GOF	Autoinflammation, hypogammaglobulinemia
STAT5B	AD	LOF	Growth hormone insensitivity syndrome (GHIS) with a broad infectious phenotype

*IRF, interferon (IFN) regulatory factor; STAT, signal transducer and activator of transcription; AD, autosomal dominant, AR, autosomal recessive; LOF, loss-of-function; GOF, gain-of-function*.

### IRF3 Deficiency and Herpes Simplex Encephalitis

A number of seminal discoveries by Casanova and colleagues described an essential non-redundant role of TLR3 signaling, including TLR3, UNC93B, TRIF, TRAF3, and TBK1, and the generation of type I IFN responses in the central nervous system (CNS) in protection against herpes simplex encephalitis (HSE) (33–37). Based on this work, our group additionally described a patient with defective IRF3 signaling and HSE (38) (Figure 3). The patient, who was a 16-year-old adolescent, suffered from a severe episode of HSE with convulsions and neurological deficits and was found to have impaired type I IFN production in response to HSV-1 and a number of viral PAMPs. More specifically, the R285Q *IRF3* mutation resulted in change from the positively charged arginine to the neutral glutamine, causing functionally defective phosphorylation, dimerization, and transcriptional activation of IRF3 (38). Since no dominant negative effect of the IRF3 variant could be demonstrated, it was concluded that the mechanism was haploinsufficiency and that the inheritance was autosomal dominant (AD) with incomplete penetrance. The causal relationship between heterozygous IRF3 deficiency and HSE was supported by the reconstitution of patient fibroblasts with wild-type (wt) IRF3, resulting in normal production of type I and III IFN in response to HSV and the TLR3 ligand Poly(IC). The infectious history of the patient, like the vast majority of previously described HSE patients with defects in TLR3 signaling pathways, was notable for no previously reported increased susceptibility to other infections, suggesting specificity in the susceptibility to HSV-1 infection and development of CNS infection (38). This very narrow infectious phenotype caused by a defect of a transcription factor, which represents a point of convergence downstream of several IFN-inducing PRRs appears surprising. However, this observation may be, at least partly, explained by the more pronounced impact of the specific R285Q *IRF3* mutation on the functional interaction with the TLR3 pathway adaptor molecule TRIF, than between IRF3 and the adaptor molecules MAVS and STING of the RIG-I and DNA sensor signaling pathways, respectively, (6, 38). Importantly, a second patient with a different IRF3 variant was subsequently described in a cohort of adult patients with HSE, providing further support for IRF3 as a genetic etiology for HSE (39). Also of relevance is a case report describing the presence of a TLR3 variant in a patient with recurrent HSV-2 meningitis (Mollaret's meningitis), thus adding another piece of data to the notion of an important role of the TLR3 signaling pathway for mounting protective IFN responses during HSV-1 and HSV2 neuroinfections (40). Importantly, this is paralleled by an earlier study showing increased susceptibility to HSV-1 infection in the brain in IRF3-deficient mice (41).

### IRF7 and IRF9 Deficiency and Severe Influenza

Despite a number of single nucleotide polymorphisms (SNP)s identified by genome wide association studies (GWAS) in patients with severe disseminated influenza infection, as well as evidence from mouse studies of an essential role of IFN in antiviral defenses against Influenza virus, the first monogenic defect associated with severe influenza was only described in 2015 (42, 43). The authors described homozygous IRF7 deficiency in a 2.5-year-old girl with severe influenza and acute respiratory distress syndrome (Figure 3). Functional studies demonstrated abolished type I IFN production in pDCs in response to Influenza A virus (IAV) and consequently elevated IAV replication in fibroblasts. The results were were further supported by including airway epithelial cells derived from induced pluripotent stem cells from the patient revealing impaired IAV replication and reduced IFN production (42).

Again the common theme is a relatively narrow infectious phenotype of the patient in contrast to *Irf7* knock-out mice which exhibit elevated susceptibility to a number of RNA- and DNA viruses (44, 45). Finally, there are some remaining pieces to the puzzle, since the infectious phenotype of patients with mutations in IRF3 and STAT1 that impair induction and responsiveness to type I IFN, respectively, have not revealed a similar increased risk of severe influenza infection (38, 46). This intriguing observation may suggest a particularly important role of the IRF3-IRF7 amplification loop for rapid production of large amounts of IFNβ as well as multiple IFNα subtypes in antiviral defense against IAV in lung tissue and/or alveolar macrophages (43).

Recently, life-threatening influenza was also reported in a child with autosomal recessive (AR) homozygous IRF9 deficiency (47) (Figure 3). Since IRF9 acts downstream of type IFN as part of the ISGF3 complex, IRF9 deficiency represents a defect in the response to IFN rather than in the induction of IFN responses. Prior to admission with severe influenza at the age of 5 years, the patient had an infectious history with several hospital admissions with RSV bronchiolitis, severe disease with biliary perforation presumably secondary to vaccination with the measles-mumps-rubella (MMR) vaccine, and frequent fevers. Based on serological evidence of infections with HSV, cytomegalovirus (CMV), rhinoviruses, and enteroviruses without particularly severe clinical infections, the report suggested a relatively narrow phenotype of IRF9 deficiency (47). The authors demonstrated normal responses to STAT1 homodimers and STAT1/2 heterodimers from a GAS promoter in contrast to impaired responses from an ISRE promoter downstream of the heterotrimer STAT1/STAT2/IRF9 (47) (Figure 3). The causal relationship between homozygous IRF9 deficiency and increased susceptibility to influenza was convincingly demonstrated by the rescue of IFN responses and control of viral replication after expression of wt IRF9 in patient cells (47). In addition to IRF7 and IRF9 deficiency predisposing to severe influenza, a *RIG-I* variant has also been described in an adult patient with severe influenza (48). Altogether, these reports demonstrate an important role of type I (and possibly type III) IFN in antiviral immunity to influenza virus in humans.

## Diseases Associated With Defective STAT Signaling

### STAT1- and IRF8 Deficiency and Mendelian Susceptibility to Mycobacterial Disease

AD STAT1 deficiency was among the first genetic etiologies of mycobacterial disease to be described (49) (Figure 3). This condition, which was termed Mendelian susceptibility to mycobacterial disease (MSMD), primarily leads to severe infection with atypical mycobacteria and the weakly virulent Bacille-Calmette-Guerin (BCG) vaccine strain. However, severe viral infections, particularly HSE, and increased susceptibility to certain intracellular bacteria, including *Listeria monocytogenes* and salmonella species, as well as fungi have also been described (50–52). The fundamental and non-redundant role of the IL12-IFNγ circuit in the intercellular communication between macrophages/dendritic myeloid cells and T/NK cells in immunity to mycobacteria has been reinforced by the reports of several defective molecules within these pathways giving rise to MSMD with a very similar phenotype. Thus, MSMD can also originate from defects in IL12 p40, IL12RB1, IFNGR1, IFNGR2, NEMO, ISG15, TYK2, CYBB, IRF8, and most recently in SPPL2a (50–63). In addition, mycobacterial infection is a prominent feature of the MonoMAC syndrome caused by *GATA2* mutations (64, 65). While various heterozygous *STAT1* mutations were described to be associated with impaired IFNγ responses (49, 66), an intriguing aspect of AD STAT1 heterozygous dominant MSMD is the apparent partial preservation of IFNα/IFNβ responses and lack of broad susceptibility to viruses, as might have been expected, given the central position of STAT1 downstream of the type I IFN receptor (29, 67, 68) (Figure 3). However, when the first patient with AR STAT1 defect, with complete loss of STAT1 function, was identified, it turned out that this phenotype indeed does show increased susceptibility to a broad range of viral infections in addition to MSMD (2, 58). In addition, patient cells are unresponsive to both IFNγ as well as to IFNα and IFNβ, and even also to IFNλ and IL27 (69). Finally, hypomorphic STAT1 alleles have been described to underlie AR STAT1 deficiency and display a milder, partial phenotype (46, 70–72).

Altogether, MSMD is a clear example of a PID with a relatively narrow infectious phenotype that may originate from a number of molecules belonging to the same functionally connected immunological pathway.

### STAT2 Deficiency and Fulminating Vaccine Strain Measles Virus Infection

Whereas STAT1 deficiency, together with other genetic abnormalities in the macrophage - T cell circuit governing IL12-IFNγ immunity, gives rise to MSMD, STAT2 deficiency was reported in 2013 to cause a narrow infectious phenotype with a fulminating disease course in a 5-year old child following MMR vaccination (73) (Figure 3). Moreover, an infant brother died from a febrile illness following a presumed viral infection of unknown etiology. Detailed studies revealed a homozygous mutation in intron 4 of *STAT2* preventing correct RNA splicing in patient cells. In addition, the authors demonstrated absence of STAT2 protein expression, significantly impaired type I IFN signaling, as well as abnormal permissiveness to viral replication in patient cells *in vitro* (73). Intriguingly however, patients with STAT2 deficiency have not been described to suffer from neither mycobacterial infection, nor a broad spectrum of viral infections, which might have been anticipated based on profoundly defective IFN responses found *in vitro*. This may suggest partial redundancy between STAT1 and STAT2 with a dominating role of STAT1 in terms of immunity to mycobacteria, i.e., in individuals with STAT1 deficiency STAT2 can partially compensate with regards to mycobacterial infection, whereas STAT1 cannot compensate for the lack of STAT2 when it comes to measles virus infection.

### STAT3 Defect and Hyper-IgE Syndrome With Prominent Infectious- and Somatic Phenotypes

Already in the initial description Hyper-IgE syndrome (HIES), originally termed Job's syndrome, “cold” abscesses caused by *Staphylococcus aureus* was described as a prominent feature (74). Other characteristics include eczematoid rashes presenting already during the neonatal period, recurrent sinopulmonary infections, skin abscesses, chronic mucocutaneous candidiasis (CMC), and eosinophilia, in combination with significantly elevated serum IgE>2,000IU/mL, and frequently even higher (75, 76). Fungal infections with *Pneumocystic jirovecii*, histoplasma, coccidioides and cryptococcus have been described to cause mucocutaneous- and gastrointestinal infections as well as meningitis. Moreover, AD HIES patients have been reported to exhibit increased susceptibility to VZV reactivation and Epstein-Barr virus (EBV) viremia (77, 78). The complexity of AD HIES pathogenesis is evidenced by the extensive list of non-immunological manifestations reported (76, 78). Abnormal craniofacial features include characteristic facies, craniosynostosis, high-arched palate, and sometimes retained childhood dentition (79, 80). Within the musculoskeletal system hyperextensibility, scoliosis, osteoporosis, and minimal trauma fractures are observed (76). Increasing awareness has also been on vascular abnormalities, including coronary artery aneurisms and hypertension (81). Similarly to many other PIDs, AD-HIES patients are at an increased risk of developing malignancies, particularly non-Hodgkin lymphoma (82).

The identification of STAT3 as the genetic origin of HIES in 2007 paved the way for a much more detailed understanding of the pathogenesis underlying the immunological abnormalities and infectious disease spectrum as well as the somatic features observed in this disease (83, 84) (Figure 4). The first study, revealed increased innate immune responses and impaired IL6 signaling, and further identified both inherited familial and sporadic mutations within the *STAT3* locus in patients with HIES (83). These *STAT3* mutations were either missense mutations or in-frame deletions and appeared to be localized primarily within the SH2-domain or the DBD of the molecule and act by a dominatint negative mechanism (83, 84). However, when mutations were present within the DBD of *STAT3*, expression, phosphorylation, and nuclear translocation of STAT3 were found to be normal compared to the situation in healthy controls (84). In contrast, in patients harboring *STAT3* mutations affecting the SH2 domain or the trans-activating domain, cellular STAT3 phosphorylation at tyrosine 705 was reduced (85). The specific mechanism, by which STAT3 mutations abolish the function of the molecule, therefore remains to be fully clarified.

The central role played by STAT3 in signaling downstream of IL6 is believed to explain a substantial part of the immunodeficiency observed in AD HIES patients (83). This notion is supported by a report describing severe staphylococcal infection in a child with anti-IL6-antibodies, providing further evidence that IL6 is critical in response to human infection with staphylococci (86). Moreover, STAT3 has been shown to negatively regulate type I IFN responses and inflammatory TLR signaling (87). A hallmark of AD HIES is the presence of impaired Th17 responses, and accordingly STAT3 mutations have been demonstrated to result in a failure of Th17 T cell differentiation (88, 89). IL17 signaling also plays a role in neutrophil proliferation and chemotaxis, possibly explaining abnormal neutrophil responses and - recruitment to lung and skin causing recurrent staphylococcal infection in these organs and tissues in HIES (90, 91). Concerning the origin of the highly elevated IgE levels characteristic of the disorder, this feature has been suggested to reflect a role for STAT3 downstream of IL21 receptor signaling, based on the observation of elevated IgE levels in mice deficient in IL21R (83, 92) (Figure 4). Notably, HIES also exists in an AR form that may be caused by defects in either TYK2, dedicator of cytokinesis (DOCK)8, or phosphoglucomutase (PGN)3, although these present with a somewhat different phenotype, including a more pronounced tendency toward cutaneous viral infections and without the somatic phenotype characteristic of AD HIES resulting from *STAT3* mutations (78, 93–95). However, TYK2 deficiency as a genetic cause of HIES remains controversial, since TYK2 deficient patients presenting with mycobacterial and viral infections in the absence of HIES have been reported (56).

### STAT5B Deficiency in Patients With Short Stature and Immunodeficiency

STAT5B plays a key role downstream of the IL2 receptor and the growth hormone receptor (GHR), explaining why STAT5B defect causes Growth hormone insensitivity syndrome (GHIS) with a complex infectious and somatic phenotype (Figure 4). This medical condition was first described by Kofoed et al. in 2003 and constitutes a syndrome with short stature, facial dysmorphism, autoimmune manifestations, and severe infections (9, 10). The spectrum of autoimmune manifestations include autoimmune thyroiditis, idiopathic thrombocytopenic purpura, lymphocytic interstitial pneumonitis, and eczema (9, 10, 96). Although STAT5A and STAT5B molecules are very similar and share a high degree of identity, they differ in both DNA binding- and transactivation domains, providing and explanation for the non-redundant roles of STAT5B in human growth and immunity. The immunological phenotype includes a reduced number of CD4^+^CD25high Foxp3^+^ cells in STAT5B patients, which is thought to contribute to the immune dysregulation of the disease (67). Indeed, studies in *Stat5a/Stat5b* double-deficient mice have demonstrated reduced numbers of Treg cells, functionally connected to the development of autoimmunity and lymphocytic infiltrations (97). Moreover, based on the immune phenotype in mice, an increased rate of T cell apoptosis has been suggested to contribute to T cell lymphopenia and the broad susceptibility to infections observed in STAT5B deficient patients (67). Finally the insensitivity to GH originates from the role of STAT5B in inducing expression of insulin growth factor (IGF)-1 following GHR activation and STAT5B phosphorylation by JAK2 (67) (Figure 4).

## Diseases Associated With Excessive STAT Signaling

### STAT1 Gain-of-Function in Th17 Deficiency and Chronic Mucocutaneous Candidiasis

A common theme in several PIDs is that different mutations in a given molecule may have rather different functional consequences and impact and hence may result in entirely different clinical pictures (3). A good example of this is the major difference between STAT1 deficiency (described above) as opposed to the disease caused by STAT1 gain-of-function (GOF) (Figures 3, 4). Two groups of investigators independently established heterozygous STAT1 GOF as a cause of Th17 deficiency and AD CMC. Van de Veerdonk et al. analyzed 5 different families constituting 14 cases of AD CMC, leading to the finding of defective production of IFNβ, IL17, and IL22 in response to candida, and the identification of heterozygous mutations within conserved residues in exon 10 encoding the CC domain of *STAT1* (98). Simultaneously, Liu et al. identified heterozygous variations in the *STAT1* gene within the CC domain by WES (16). Functional analyses of the mutant alleles revealed GOF mutations by a mechanism involving impaired nuclear dephosphorylation of Stat1, and indeed, nuclear dephosporylation rather than cytosolic hyperphosphorylation may be the dominant molecular mechanism underlying the immunological abnormalities in STAT1 GOF (99). Altogether, several different amino acid changes have been reported to cause either STAT1 LOF or GOF, most prominently in the CC domain and the DB domain of the molecule, although the precise functional impact of individual mutations may be difficult to estimate by bioinformatics alone, but requires mutagenesis studies (89, 98–104). Moreover, autoimmunity is a common feature of this patient population. Thyroid disease, enteropathy, alopecia, autoimmune cytopenias, type I diabetes and systemic lupus erythematosus-like disease have been reported (105). More recently, progressive multifocal leukoencephalopathy (PML) caused by reactivation of JC virus was described in a small number of patients with STAT1 GOF, indicating a profound T cell deficiency in this condition (98).

Several hypotheses have been presented to explain the molecular mechanism, whereby *STAT1* GOF impairs the development of Th17 cells and IL17 responses (89). One of these states that STAT1 counteracts the gene expression induced by STAT3 downstream of Th17 cell differentiating- and generating signals, such as IL6, IL21, and IL23 signaling (106) (Figure 4). Another idea is that exaggerated IFNα/β- and IL27 responses inhibit the development of the Th17 subset of T cells (67, 107). However, the precise molecular mechanisms behind impaired STAT3 signaling in these patients remains unknown. As to the pathogenesis of the autoimmune phenomena sometimes associated with STAT1 GOF, this is not readily explained by decreased numbers of Treg cells, which appear to be normal (108). However, based on the observation that some of the features in STAT1 GOF patients overlap with the group of monogenic diseases termed interferonopathies, including an elevated IFN signature, i.e., upregulation of ISGs in the blood, this may account for some aspects of the autoimmunity present (109–111).

### STAT3 GOF in an Autoinflammatory Phenotype With Hypogammaglobulinemia and Lymphoproliferation

Defective STAT3 has been described above as the genetic origin of AD HIES. However, more recently it has been appreciated that the opposite, namely *STAT3* GOF mutations, can cause early-onset lymphoproliferation and autoimmunity (3, 112–114) (Figure 4). This severe pleiotrophic phenotype with multiorgan involvement encompasses hypogammaglobulinemia without fulfilling the criteria for common variable immunodeficiency (CVID), together with autoimmune cytopenias, lymphocytic interstitial pneumonia, enteropathy, hepatitis, and arthritis. Examination of the immunological phenotype revealed hypogammaglobulinemia, T cell lymphopenia (with increased fraction of double-negative T cells), impairment in switched memory B cells, decreased pDCs, as well as reduced regulatory T cells, in accordance with autoimmunity as a dominating feature (77, 112–114).

## Prophylaxis and Treatment of Diseases Affecting IRFs and STATs

A number of prophylactic and therapeutic strategies are currently available to prevent and treat PIDs in general, including those involving IRFs and STATs. Overall, since these conditions are generally severe and life threatening and often diagnosed early in life, they need correction by bone marrow transplantation (BMT) or hematopoietic stem cell transplantation (HSCT), where results are generally excellent, particularly in the case of early treatment, although depending on the underlying condition and genetic etiology. Thus, survival and cure is currently reached in up to 90% of patients undergoing HSCT for severe PIDs (115, 116). A prerequisite for these positive results, however, is early diagnosis and HSCT before secondary complications develop. This goal may be achieved in the future by introducing advanced programs for newborn screening for some of the major PIDs, which has proven possible in the case of SCID, even in developing countries (117, 118). Among the PIDs related to IRF and STAT deficiencies covered in the present review, HSCT are used for MSMD, HIES, GHIS as well as for STAT1 GOF and STAT3 GOF (119).

I addition, general vaccination strategies are relevant in the case of severe influenza in IRF7 and IRF9 deficiency. Prophylaxis with antibiotics (for example co-trimoxazole) and antifungals (such as intraconazole) are broadly employed with good results in conditions, such as HIES, CMC, GHIS, and MSMD (119). Prophylactic aciclovir treatment may be recommended in some cases of HSE caused by IRF3 deficiency, at least in recurrent cases. In many conditions dominated by abnormalities in B cell development and -responses with resulting hypogammaglobulinemia, immunoglobulin substitution therapy is a cornerstone of maintenance prophylaxis against infection with many years of experience and good evidence of beneficial effects in various PIDs (115, 119).

Novel medications include use of selective JAK inhibitors for the treatment of excessive production of type I IFN in the group of interferonopathies caused by mutations in STING or other molecules within the DNA sensing pathways (111, 120). Such approaches may also be relevant in a number of the described PIDs, such as STAT1 GOF and HIES, in which type I IFN is believed to play a role in generating the autoimmune state. On the other hand, HSE associated with impaired antiviral type I IFN responses might be treated with type I IFN as an adjunctive to the current standard therapy with antiviral aciclovir, although large clinical trials are ongoing but need to prove efficacy of this treatment approach.

Finally, looking into the future, new powerful technologies, such as CRISPR/Cas editing of genetic defects in PIDs is a promising avenue, which has already demonstrated potential success for medical conditions such as β-globin expression in thalassemia, and more recently in the case of certain PIDs, most notably chronic granulomatous disease (CGD) and adenosine deaminase (ADA)-severe combined immunodeficiency (SCID) (121–124). It seems highly likely that the CRISPR/Cas technique will be more widely applicable, also to some of the PIDs described here involving IRFs and STATs. Among these STAT1 and STAT3 deficiency in MSMD and AD HIES might be good candidates for gene correction.

## Conclusion and Future Perspectives

Lessons learned from the fascinating decade-long unraveling of PIDs are numerous and have provided fundamental new insight into basic infection immunology and the correlates of protective immunity against bacterial, viral, and fungal pathogens in humans. More recently, it has also brought new knowledge about the pathogenesis of a number of conditions with underlying autoimmunity and autoinflammation. Importantly, these discoveries have been taken directly into clinical medicine and are thus the basis, upon which the invention and development of specific prophylactic and therapeutic strategies for patients are based.

Notably, this journey has repeatedly demonstrated significant differences between mouse and human immunity, which underscores the importance and strength of studying human patients to understand basic innate and adaptive immunology in humans. Moreover, with the increasing awareness of the major contribution of autoimmunity and autoinflammation as part of many PIDs, there is now a need for the development of approaches to treating these complex pathologies (114). Again, this reinforces the essential need for understanding basic molecular and cellular mechanisms underlying these states of dysregulated immunity.

In conclusion, improved understanding of the genetic defects and immunopathogenesis of various currently known PIDs is of fundamental importance to identify new targets and immunomodulatory agents in future management of conditions of immune dysregulation, infection, and inflammation. Given that this field of research is in a rapidly expanding phase, it is to be anticipated that many additional human inborn errors of immunity will be uncovered within the next few years. Finally, despite the extensive list of PIDs caused by mutations in IRFs and STATs as described in the present review, we are likely to learn even more in the future, thus providing new insights into both basic biology, signaling, and regulation of these transcription factors and the human pathologies their dysregulation may cause.

## Author Contributions

The author confirms being the sole contributor of this work and has approved it for publication.

### Conflict of Interest Statement

The author declares that the research was conducted in the absence of any commercial or financial relationships that could be construed as a potential conflict of interest.
